
JOURNAL INFORMATION
==============================
NLM Title Abbreviation: Wirtschaftsdienst
Journal ID: Wirtschaftsdienst
NLM Journal ID: 101086612

Wirtschaftsdienst (Hamburg, Germany : 1949)

ISSN: 0043-6275

ARTICLE INFORMATION
==============================
PMCID: PMC7896177
PMID: 33642640
DOI: 10.1007/s10273-021-2838-0
Article ID: 2838
Article version: 1
Subjects: Leitartikel

Gesellschaftliche Akzeptanz als Schlüssel in der Corona-Pandemie

Social Acceptance as Key in the Corona Pandemic

Fratzscher Marcel MFratzscher@diw.de

grid.8465.f 0000 0001 1931 3152 Deutsches Institut für Wirtschaftsforschung, Mohrenstr. 58, 10117 Berlin, Deutschland
Electronic publication date: 2021 Feb 20
Print publication date: 2021
Volume: 101
Issue: 2
First page: 70
Last page: 71
Copyright: © Der/die Autor:in(nen) 2021
License: Open Access: Dieser Artikel wird unter der Creative Commons Namensnennung 4.0 International Lizenz veröffentlicht (http://creativecommons.org/licenses/by/4.0/deed.de).
Open Access wird durch die ZBW–Leibniz-Informationszentrum Wirtschaft gefördert.
License URL: https://creativecommons.org/licenses/by/4.0/

issue-copyright-statement: © The Author(s) 2021

==============================

Die öffentliche Debatte um die Strategie im Umgang mit der Pandemie hat ein Maß an Emotionalität erreicht, das zu einem Kontrollverlust in der Krise führen kann. Viele Wissenschaftler:innen beteiligen sich mit Vorschlägen, wie die Politik ihre Strategie ändern sollte. Ein solcher Diskurs ist wichtig für eine Demokratie. Aber er muss auch realistisch bleiben, um Lösungen für akute Probleme zu liefern. Dabei ignorieren Vorschläge aus den Wirtschafts- und Sozialwissenschaften häufig den wichtigsten Faktor für den Erfolg einer jeden Strategie: die gesellschaftliche Akzeptanz. Zu einer sachlichen Debatte gehört nicht nur die Identifikation der Fehler, sondern auch der Erfolg. Hier kann Deutschland punkten: Die erste Pandemie-Welle wurde vergleichsweise gut bewältigt, da die Politik früh und konsequent reagierte und klar kommunizierte. Zudem hat die überwiegende Mehrheit der Menschen die getroffenen Maßnahmen unterstützt und befolgt (Fratzscher, 2020).

Auch bei der nun viel kritisierten Impfstrategie hat die Politik vieles richtig gemacht: der europäische Ansatz, die gründliche wissenschaftliche Prüfung und klare Prioritäten bei Impfungen haben Zuverlässigkeit, Vertrauen und Solidarität in den Mittelpunkt gestellt. Die Verantwortung für die geringe Verfügbarkeit von Impfdosen Anfang 2021 liegt somit nicht bei dem, was Athey et al. (2020) als Teil ihrer Strategie des Advance Market Commitment (AMC) bereits im Mai 2020 den Pull-Faktor nennen – eine signifikante Nachfrage zu angemessenen Preisen von verschiedenen Anbietern, um die Entwicklung des Impfstoffs zu beschleunigen. Dies hat die EU im Großen und Ganzen richtig gemacht. Der Fehler lag vielmehr beim Push-Faktor: Man hat ignoriert, dass die Pharmakonzerne selbst keine Anreize haben, durch eine starke und kostspielige Erhöhung ihrer Kapazitäten den Impfstoff früher zur Verfügung zu stellen. Der Staat hätte selbst im Sommer 2020 mit massiven Investitionen in Kapazitäten und einer Preisgestaltung, mit höheren Preisen für eine frühere Lieferung agieren müssen (Fratzscher, 2021). Gleichzeitig sind Forderungen falsch, der Staat könne mit einer AMC-Strategie noch in den kommenden Monaten ein deutlich höheres Angebot an Impfstoffen schaffen. Ein Aufbau an Kapazitäten dauert bis zu zwölf Monate. Der Eindruck, der Staat könne kurzfristig zusätzliche Kapazitäten schaffen, läuft Gefahr, das Vertrauen in die Politik weiter zu erodieren. Zudem wird damit ein Impfnationalismus befeuert, bei dem es darum geht, welche Nation mehr vom knappen Impfstoff erhält.

Die Realität ist, dass wir in Deutschland und Europa zumindest bis Ende des Sommers 2021 ohne ausreichende Impfungen mit dem Virus leben müssen. Der Fokus der gegenwärtigen Debatte sollte daher vielmehr beim Umgang mit dem Virus und dem Lockdown liegen. Auch in dieser Frage laufen die Empfehlungen weit auseinander. Auf der einen Seite stehen die Verfechter von No-Covid-Strategien (Baumann et al., 2021), die auf eine massive Reduzierung der Inzidenz abzielen. Auf der anderen Seite stehen die Befürwortenden einer weicheren Strategie, die auf eine baldige Lockerung der einschneidenden Maßnahmen, vor allem der Schließung von Kitas und Schulen drängen (Grewenig et al., 2020). Beide Positionen haben überzeugende Argumente: eine starke Begrenzung der Zahl der Neuinfizierten ermöglicht einen nachhaltigen Neustart der Wirtschaft und Planungssicherheit für Bevölkerung und Unternehmen. Die Befürwortenden der gegengesetzten Strategie weisen zu Recht auf den massiven Schaden einer restriktiven Strategie vor allem für die verletzlichsten Menschen in der Gesellschaft hin. Zudem unterschätzen die Verfechter von No Covid, dass ihre Strategie mit hoher Wahrscheinlichkeit scheitern und damit kontraproduktiv sein könnte. Zum einen, weil eine No-Covid-Strategie auf Inseln oder Halbinseln funktionieren mag, aber nicht in einem dicht besiedelten Land wie Deutschland mit vielen Ländergrenzen und häufig fließenden Übergängen zwischen Städten. Zum anderen würde die No-Covid-Strategie auch deshalb scheitern, weil sie harte Restriktionen für einen sehr langen Zeitraum erfordern würde, die von den Menschen in Deutschland nicht mitgetragen werden. Wenn aber die Akzeptanz fehlt und Menschen sich nicht mehr an die Regeln halten, dann verlieren Politik und Gesellschaft die Kontrolle über die Pandemie und der Schaden wird sehr viel höher sein als gegenwärtig. Die jetzige Strategie der Politik ist viel besser als ihr Ruf. Sie versucht, eine Balance zwischen dem Schutz von Gesundheit, Gesellschaft und Wirtschaft unter Wahrung von Grundrechten und Freiheiten zu finden, die eine möglichst breite Unterstützung erfährt. Diese Strategie hat sich im Frühjahr und Sommer 2020 als richtig erwiesen. Der Grund für das Scheitern, die zweite Infektionswelle früher zu stoppen, liegt somit nicht in der Strategie per se, sondern in der zu zögerlichen und inkonsequenten Umsetzung der Maßnahmen. Wichtig ist eine deutliche Verbesserung beim Testen, Nachverfolgen und Isolieren, um die zweite Welle dauerhaft unter Kontrolle zu halten und eine dritte Welle zu verhindern. Im Januar 2021 konnte laut Robert-Koch-Institut bei mehr als 80 % der Infizierten die Kontaktperson nicht identifiziert werden. Durch regelmäßiges Testen in Schulen und an Arbeitsplätzen könnten Ansteckungsketten unterbrochen werden.

All dies bedeutet, dass ein schnelles Ende der Pandemie nicht abzusehen ist und wir wohl zumindest den größten Teil des Jahres 2021 weiter mit dem Virus leben müssen. Die Szenarien für die Konjunkturentwicklung sind ganz unterschiedlich, der erhoffte Aufschwung im laufenden Jahr ist keine ausgemachte Sache (DIW Berlin, 2020). Der IWF (2020) schätzt, dass ein anhaltender Lockdown oder ein stetiges Lockern und Verschärfen von Restriktionen das Wachstum der Weltwirtschaft um 4 Prozentpunkte 2021 im Vergleich zu einem Szenario ohne starke zweite Infektionswelle reduzieren könnte. Damit ist auch ein Schrumpfen der deutschen Wirtschaft 2021 ein mögliches Szenario. Deshalb kann und sollte sich die Wirtschaftspolitik auch auf ein Szenario einer anhaltenden Rezession vorbereiten (Clemens et al., 2020). Im internationalen Vergleich gibt es kaum ein Land, das größere Wirtschaftshilfen und Konjunkturprogramme aufgelegt hat als Deutschland (OECD, 2020). Ein frühzeitiges Signal der Politik an Unternehmen und Beschäftigte, dass sie ihre Wirtschaftshilfen – vor allem die Überbrückungshilfen für Unternehmen und das Kurzarbeitergeld für Beschäftigte – weiter verlängern, wie auch Hilfen für Familien, Solo-Selbstständige, Minijobber:innen und andere vulnerable Gruppen, sind wichtige wirtschaftspolitische Maßnahmen, um zumindest ein Minimum an Stabilität zu gewährleisten. Die neuen Maßnahmen von Anfang Februar 2021 sind ein erster Schritt in diese Richtung, weitere müssen folgen – so auch ein Investitionsprogramm, das weit über das Ende der Pandemie hinaus einen wirtschaftlichen Impuls verspricht. Nun muss es auch in der zweiten Welle um die konsequente Umsetzung akzeptierter Maßnahmen gehen, mit einem starken Gewicht auf besserem Testen, Nachverfolgen und Isolieren.

Marcel Fratzscher
Deutsches Institut für Wirtschaftsforschung
mfratzscher@diw.de

Marcel Fratzscher ist Hochschullehrer an der Humboldt-Universität zu Berlin und Präsident des Deutschen Instituts für Wirtschaftsforschung (DIW) in Berlin.


Literatur

Athey, S., M. Kremer, C. Snyder und A. Tabarrok (2020), In the Race for a Coronavirus Vaccine, We Must Go Big. Really, Really Big, New York Times, 4. Mai.
Baumann, B. et al. (2021), Eine neue proaktive Zielsetzung für Deutschland zur Bekämpfung von SARS-CoV-2, Die Zeit, 18. Januar, http://zeit.de/wissen/gesundheit/2021-01/no-covid-strategie.pdf (8. Februar 2021).
Clemens, M., G. Dany-Knedlik, S. Junker und C. Michelsen (2020), „Harter“ Lockdown infolge der zweiten Corona-Welle: Deutsche Wirtschaft wächst 2021 deutlich weniger stark, DIW aktuell, 57, Dezember.
DIW Berlin (2020), Weltwirtschaft: Erholung ausgebremst: Grundlinien der Wirtschaftsentwicklung im Winter 2020, DIW Wochenbericht, 50, Dezember.
Fratzscher, M. (2020), Die neue Aufklärung–Wirtschaft und Gesellschaft nach der Corona Krise, Berlin Verlag, Oktober.
Fratzscher, M. (2021), Das Impfdesaster, an dem wir alle Mitschuld tragen, Spiegel Online, 6. Januar.
Grewenig, E., P. Lergetporer, K. Werner, L. Wößmann und L. Zierow (2021), „Corona-Schulschließungen treffen leistungsschwächere Schüler*innen besonders hart“, Ökonomenstimme, 21. Dezember.
IWF (2020), World Economic Outlook, Oktober 2020.
OECD (2020), Turning hope into reality, Economic Outlook, Dezember.
